# Diagnosis, Management, and Outcome of Antituberculous Drug–Induced Liver Injury in a Low‐Resource Setting: A Case Report

**DOI:** 10.1155/crdi/1056322

**Published:** 2026-07-29

**Authors:** Samuel Alomatu, Mirabel Kah-Keh Nanjoh, Salimatu Asam Moro, John Gerald A. Adiiboka, Pascal Deeshini Aliu Alhassan, Odalys Rivera Hernandez

**Affiliations:** ^1^ Department of Internal Medicine, Tamale Teaching Hospital, Tamale, Ghana, tamaleteachinghospital.org; ^2^ Department of Public Health, Faculty of Medicine and Health Sciences, Walter Sisulu University, Mthatha, South Africa, wsu.ac.za; ^3^ School of Medicine, University for Development Studies, Tamale, Ghana, uds.edu.gh

**Keywords:** antituberculosis drugs, case report, drug-induced liver injury, low- and middle-income countries

## Abstract

**Background:**

Antituberculous drugs carry substantial hepatotoxic risk, most commonly manifesting as idiosyncratic drug–induced liver injury. Although cases of antituberculosis drug–induced liver injury (AT‐DILI) have been reported in Ghana, significant challenges remain in its diagnosis, monitoring, and management in low‐resource settings.

**Main Symptoms and Clinical Findings:**

We report the case of a 50‐year‐old woman who developed severe AT‐DILI, initially misdiagnosed as cholecystitis, on a background of chronic hepatitis C infection with established liver cirrhosis. She was referred to the Tamale Teaching Hospital during the continuation phase of first‐line antituberculosis therapy, presenting with nausea, anorexia, and right upper quadrant abdominal pain. A clinical examination revealed mild pallor, jaundice, Grade 1 bilateral pitting pedal edema, and moderate ascites.

**Diagnosis, Therapeutic Intervention, and Outcome:**

At the referral facility, diagnostic and monitoring constraints were encountered, including the unavailability of liver biopsy, the lack of routine baseline liver function testing, and out‐of‐pocket costs for serial laboratory monitoring. In addition, the absence of single‐drug formulations of isoniazid and rifampicin precluded sequential rechallenges, necessitating rechallenges with a fixed‐dose combination during the continuation phase. The patient failed rechallenge and was transitioned to an all‐oral second‐line antituberculous regimen consisting of bedaquiline, pretomanid, linezolid, and moxifloxacin, alongside direct‐acting antiviral therapy for chronic hepatitis C. This resulted in the resolution of liver injury and the successful completion of tuberculosis treatment without complications.

**Conclusion:**

This case highlights the diagnostic and management challenges of AT‐DILI in low‐resource settings, particularly where baseline liver function testing, serial monitoring, and access to single‐drug formulations for rechallenge are limited.

## 1. Introduction

The number of persons on antituberculosis drugs who develop drug‐induced liver injury is shown to be increasing over time, with a pooled prevalence of 11.5% [1], and country‐specific incidence variability ranging from 41.97% in Thailand [2], 15% in Ghana [3], 12.6% in India [4], 7.9% in Ethiopia [5], and 5.4%–10.7% in China [6, 7]. The occurrence of antituberculosis drug‐induced liver injury (AT‐DILI) interrupts the tuberculosis treatment course and challenges patients’ adherence, thus increasing the risk of drug resistance and poor outcomes [8, 9]. In Ghana, the incidence of tuberculosis is 126 per 100,000, which is responsible for 27 per 100,000 deaths among HIV uninfected people [10].

The antituberculosis agents used in Ghana are globally recommended [11] and include isoniazid, rifampicin, pyrazinamide, and ethambutol as key first‐line drugs, over a 6‐month course for an expected pathogen‐free diagnostic outcome. During the first 2 months of the treatment, isoniazid, rifampicin, and pyrazinamide, combined with ethambutol, are administered, followed by 4 months of rifampicin and isoniazid [11].

However, these drugs are known to carry substantial hepatotoxic risks, manifesting as drug‐induced liver injury, predominantly idiosyncratic [7, 12], albeit hepatic adaptation when reintroduced after DILI. An increased risk of AT‐DILI has been found with hepatitis B and C virus coinfection, female, HIV‐infection, older age, malnutrition, underweight body mass index (BMI), alcohol consumption, low albumin, statin coadministration, and slow N‐acetyltransferase 2 acetylator (NAT2) genotype [1–3, 5, 6, 13–15]. Even though the mechanisms of AT‐DILI are not fully understood, metabolites produced by some of these drugs have been shown to cause DILI. For example, isoniazid releases acetyl diazine and reactive acetyl free radicals following metabolism by NAT2 and can easily be converted to INH hydrazine, which contributes to liver injury [4].

Although symptoms of AT‐DILI are nonspecific and might mimic symptoms of active tuberculosis, reported symptoms include fever, rash, fatigue, loss of appetite, and dark brown urine, or eosinophilia [9]. Diagnosis of AT‐DILI is often established based on elevated liver enzymes [16, 17], with a large body of evidence of its application in the available literature [2, 8, 9]. Guidelines for diagnosing AT‐DILI recommend several criteria, including baseline and continuous monitoring of liver function tests (LFTs) among high‐risk persons [8, 18, 19]. In resource‐constrained settings like Ghana, already overburdened with high TB rates, declining cure rates, and substantial TB‐related death rates [10, 20, 21], AT‐DILI poses a significant challenge, particularly in identifying and managing cases.

This article aims to report a case of AT‐DILI at the Tamale Teaching Hospital, analyze the presenting symptoms and hematological manifestations, and discuss the management outcomes and challenges encountered in diagnosing and managing the case. The CARE checklist and writing outline aided in developing this case report [22].

## 2. Case Presentation

A 50‐year‐old woman was referred to the Tamale Teaching Hospital in the Northern Region of Ghana from a district‐level hospital where she had been diagnosed with pulmonary tuberculosis, rifampicin susceptible, and initiated on the first‐line fixed‐dose antituberculous drugs. She had completed the 2‐month intensive phase and was on the continuation phase of isoniazid and rifampicin fixed‐dose combination for 14 days before presentation. She presented to the referral facility with a 9‐day history of right upper quadrant pain that was of gradual onset and dull in character. It was associated with nausea and anorexia.

She had no history of alcohol intake, over‐the‐counter medications, herbal or illicit drugs, or any other prescribed medications. She was referred with this history to Tamale Teaching Hospital for management with suspicion of cholecystitis.

She was mildly pale, icteric, afebrile, and had Grade 1 bilateral pitting pedal edema. The examination of her abdomen revealed abdominal distension with right hypochondriac tenderness and a positive shifting dullness. Her respiratory, cardiovascular, and nervous system examinations were unremarkable.

Her LFT revealed markedly elevated alanine transaminase (ALT) at 437.5 U/L, which was 12.9 times the upper limit of normal (ULN; 0–34). Total bilirubin was 101.9 μmol/L, 4 times the ULM (2–25). Her R factor calculated was 19.2, indicating hepatocellular liver injury. She was found to be hepatitis C positive with hepatitis C virus polymerase chain reaction (HCV PCR) of 5049 RNA copies/mL. Her international normalized ratio (INR) was 3.12.

She tested negative for human immunodeficiency virus via ELISA and nonreactive for Hepatitis B. Her renal function tests were normal, and her full blood count revealed normocytic anemia (hemoglobin of 9.8 g/dL, white blood cell count of 4.02, and platelets of 275). There were challenges in getting her to undergo hepatitis A immunoglobulin M (HepA IgM) and serum‐ascitic albumin gradient testing due to financial constraints along her path, as these tests are costly in low‐ and middle‐income countries (LMICs).

An ascitic tap, however, revealed a serous ascitic fluid. An abdominal ultrasound revealed features suggestive of early liver cirrhosis with moderate ascites. A presumptive diagnosis of severe AT‐DILI and liver cirrhosis secondary to chronic hepatitis C was made. Her antituberculous drugs were held, and she was put on a holding regimen of levofloxacin, ethambutol, and linezolid. She was admitted for close monitoring. Serial monitoring of ALT and bilirubin every 3 days was requested, and a plan to rechallenge her with the first‐line antituberculous drug once ALT < 100 U/L and total bilirubin < 40 μmol/L, or with a downward trend in total bilirubin.

However, due to the patient’s financial constraints, there were lapses in the frequency of LFTs. Subsequently, only ALT was used for monitoring. The ALT and bilirubin results are presented in Table 1 and reveal a downward trend after stopping isoniazid and rifampicin.

**TABLE 1:** Alanine transaminase and bilirubin results after stopping the isoniazid and rifampicin.

Date	Test results/normal range	
	Alanine transaminase (U/L)/0–34 U/L	Total bilirubin (μmol/L)/2–25 μmol/L
03/02/25	437.5	101.9
09/02/25	213.4	57.2
14/02/25	116.7	55.8
17/02/25	30.6	The patient could not afford payment

When her ALT level was below 100 U/L and total bilirubin was decreasing, the patient was rechallenged with the isoniazid and rifampicin combination the following day, 18/02/25. This was necessitated by the unavailability of single‐tablet formulations of isoniazid and rifampicin at the time, which is common in most LMICs. The rechallenge was stopped because she started vomiting during rechallenge and slowly rising ALT levels from 30.6 to 43 and 46, respectively (Table 2).

**TABLE 2:** Alanine transaminase and bilirubin results after rechallenge with isoniazid and rifampicin.

Date	Test results/normal range	
	Alanine transaminase (U/L)/0–34 U/L	Total bilirubin (μmol/L)/2–25 μmol/L
20/02/25	43	The patient could not afford payment
23/02/25	46	The patient could not afford payment

Since it was difficult to identify the single offending agent, both isoniazid and rifampicin were discontinued, and the patient was initiated on second‐line antituberculous therapy consisting of an all‐oral regimen of bedaquiline, pretomanid, linezolid, and moxifloxacin (BPALM), as there were no contraindications to its use. Oral pyridoxine was added to prevent linezolid‐associated peripheral neuropathy. A baseline electrocardiogram (ECG) prior to BPALM initiation was normal, and there were no clinical signs of peripheral neuropathy on examination. Following initiation of the BPALM regimen, the patient’s clinical prognosis was favorable, with resolution of symptoms, normalization of liver enzymes, and no recurrence of hepatic injury during follow‐up.

**FIGURE 1:**
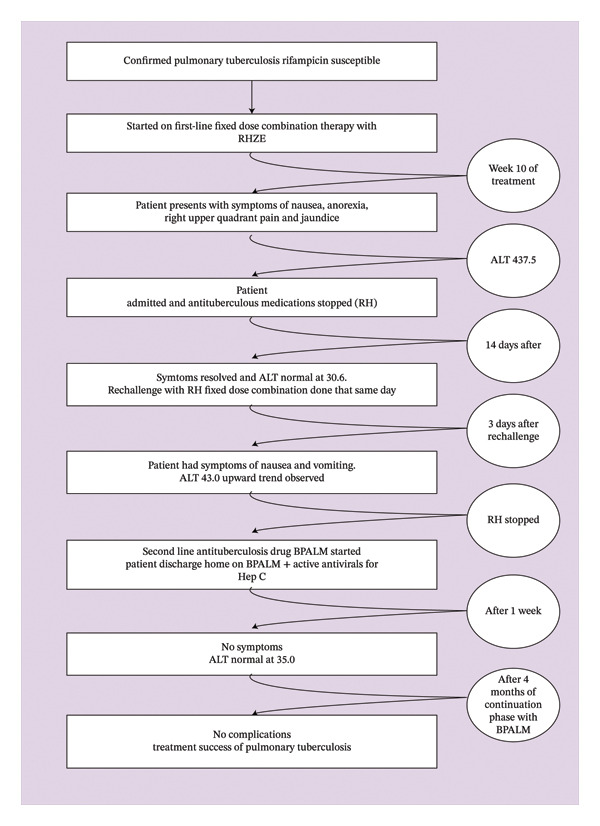
Flow diagram of the events.

She was additionally commenced on oral sofosbuvir, velpatasvir, and ribavirin for treatment of chronic hepatitis C, as well as spironolactone. She was discharged 25 days after admission and scheduled for follow‐up at 1 week with repeat ALT testing and at 2 weeks with a full blood count. At 1‐week review, she reported no nausea or vomiting, and her ALT level was 35 U/L while on BPALM. She continued BPALM for treatment of pulmonary tuberculosis, remained adherent throughout therapy, and achieved treatment success without complications or adverse effects (Figure 1 and Table 3).

**TABLE 3:** Summary of flow of events from admission to discharge at Tamale Teaching Hospital.

Date	Event	Key findings/intervention
03/02/2025	Presentation at our tertiary facility	Right upper quadrant pain, jaundice, nausea, and pedal edema ALT 437.5U/L TBIL 101.9 μmol/L, INR 3.12 HCV Positive, HCV RNA+
03/02/2025	Initial management	First‐line TB drugs stopped (started holding regimen started), and serial LFT monitoring
09/02/2025	LFT	ALT 213.4 U/L TBIL 57.2 μmol/L
14/02/2025	LFT	ALT 116.7 U/L TBIL 55.8 μmol/L
17/02/2025	ALT	ALT 30.6 U/L TBIL not done because of financial challenges
18/02/2025	Rechallenge	Fix dose INH + RIF rechallenge started, single‐drug formulation not available at the facility
20–23/02/2025	Failed rechallenge	Vomiting started, and ALT rose to 46 U/L; hence, the rechallenge was stopped
Late Feb 2025	Alternate (second line regimen BPALM)	Individualized all oral second‐line regimen commenced, and treatment for hepatitis C
Late Feb 2025	Discharged home	No symptoms, discharged home, counseled, and for review with ALT in a week
03/03/2025 (1 week postdischarge)	Follow up	No symptoms, ALT 35 U/L
June 2025	Completion of TB treatment with BPALM	Completion of the month continuation phase with BPALM with success and no complications or adverse effects

## 3. Discussion

Our patient was initially thought to have cholecystitis, as documented on the referral letter. Again, we did not have her baseline LFT before she started the antituberculous medications.

There are challenges in diagnosing and managing antituberculous DILI in Ghana. Although DILI is the most common adverse reaction of antituberculous therapy, searches done on PubMed, Google Scholar, and Research Gate showed very limited data on DILI and especially AT‐DILI in Ghana. AT‐DILI is a common reason for hospital admission and is reported to occur in 5%–33% of all adult patients with tuberculosis [8]. It accounts for about 40% of all DILI‐related hospital admissions [23].

Though we did not have her baseline LFTs, her hepatocellular pattern of liver injury with R factor of 19.2, ALT greater than 10‐fold the ULN, and total bilirubin > 40 μmol/L, regardless of her symptoms of abdominal pain, nausea, and jaundice, met the criteria for the presumptive diagnosis of AT‐DILI:

1. ALT > 120 IU/L or ALT > 3 × ULN with:
  - symptoms (vomiting, nausea, abdominal pain, encephalopathy, and bleeding) or
  - Jaundice or
  - total bilirubin > 40 μmol/L
2. ALT > 200 IU/L or ALT > 5 × ULN regardless of symptoms or bilirubin
3. ALT > 2 X baseline in patients with existing chronic liver disease, or if the patient had
 A baseline ALT > 120 U/L before antituberculous therapy

Withdrawing the offending agents showed a declining trend of ALT and total bilirubin, which aligns with the presumptive diagnosis [8].

Multiple risk factors for AT‐DILI were identified in this patient: female sex, 50 years of age, coinfection with hepatitis C, and pre‐existing chronic liver disease [8]. Other known risk factors include concomitant alcohol consumption, low BMI, drug interactions, and the isoniazid metabolism acetylator status of the patient [24].

AT‐DILI typically occurs within the first 2 months after initiation of therapy, with the highest incidence within the first 2 weeks. The onset of her symptoms after about 2 months of first‐line antituberculous drugs suggests an idiosyncratic pattern of DILI [24, 25]. Her AT‐DILI was classified as a severe form, as INR was greater than 1.5, and with symptoms [8].

Though about 90% of patients are successfully rechallenged, rechallenge has inherent challenges and is reserved for essential drugs [8, 26]. In our setting, the ideal rechallenge steps with single drug forms of isoniazid and rifampicin were not practicable since the single‐dose tablets were not available. A rechallenge with the isoniazid and rifampicin combination was used since the patient was now in the continuation phase of pulmonary TB treatment. Considering the risk for fulminant hepatitis and death, the rechallenge was stopped on noticing a rise in ALT from 30.6 gradually to 46 over 6 days [8, 26] since she was vomiting but could not afford routine monitoring. Moreover, one could not tell which single drug was implicated since she was rechallenged with a fixed dose combination as her pulmonary tuberculosis was rifampicin‐susceptible. Second‐line antituberculous drugs were started, which were better tolerated.

Perhaps the diagnosis could have been made earlier for this patient. Again, the single offending agent could have been identified if we had individual drugs at the facility. Even though during her workup for AT DILI, since it is a diagnosis of exclusion, she was found to have chronic hepatitis C, the rapid decline in her liver enzymes, especially ALT to normal values before initiation of her treatment for Hepatitis C, made us suspect this pattern of liver injury that was less likely to be from chronic hepatitis C with liver cirrhosis.

The primary takeaway lesson is to assess patients with drug‐susceptible tuberculosis on treatment for signs and symptoms of DILI at the time of initiation of treatment, during their follow‐up visits while on treatment for tuberculosis, and also anytime they get hospitalized, a LFT should be done as soon as there is any symptom or sign of DILI. Also, high‐risk patients for AT‐DILI should be counseled on symptoms of AT DILI, such as vomiting, abdominal pain, or jaundice, and the need to report immediately to a healthcare facility for assessment. There is also a need for clinicians at all healthcare facilities in LMICs to maintain a high index of suspicion for AT DILI, especially in high‐risk groups. Additionally, because tuberculosis is highly prevalent in Ghana and treatment, which is often associated with drug‐related adverse effects, is unavoidable, single‐pill fixed‐dose combinations of firstline antituberculous medications are particularly important for supporting safer and more effective therapy. Efforts to ensure the availability of these formulations in Ghana should therefore be prioritized. Ongoing efforts to end TB, such as increased case detection, improved diagnostic access, the use of TB preventive therapy, and the constant availability of medications, should be prioritized as national policy. In our case, the absence of baseline LFT and no access to liver biopsy is a limitation to the definitive diagnosis of AT DILI.

## 4. Conclusion

This case highlights how AT‐DILI can be challenging to diagnose and manage in resource‐limited settings, when baseline LFT, serial monitoring, and single‐drug formulations for rechallenge are not available. In high‐risk patients, early symptom recognition, immediate liver function testing when symptoms occur, and access to single drug formulations for sequential challenge are very critical. When first‐line drug rechallenge is not feasible or not tolerated, individualized second‐line drugs may be used as an option.

## Author Contributions

All the authors contributed to the conception, design, drafting, and revision of the manuscript.

## Funding

No funding was required nor sought to analyze and report this case.

## Disclosure

All authors read and approved the final manuscript.

## Consent

Verbal informed consent for publication was obtained from the patient. The case report does not contain any patient identifiers, and all efforts have been made to ensure patient confidentiality.

## Conflicts of Interest

The authors declare no conflicts of interest.

## Patient Perspective

The patient’s perspective on the treatment received is not included.

## Data Availability

Data will be made available upon request.
